# Elegant Medical Preparations

**Published:** 1872-03

**Authors:** 


Medicines Received.—We have been using for some time preparations from the house of Wm. R. Warner & Co., Druggists, Philadelphia, and can recommend them to the profession as both elegant and reliable. We have given their sugar coated pills and granules a thorough trial and have in every case been perfectly satisfied with their aetion.

We cannot speak from personal experience concerning their truly elegant fluid extracts, but from the high standard which the house occupies do not hesitate to recommend them as truthfully representing the qualities of the drug.

